# Trends in the epidemiology of young-onset colorectal cancer: a worldwide systematic review

**DOI:** 10.1186/s12885-020-06766-9

**Published:** 2020-04-06

**Authors:** Khalid Saad El Din, Jonathan M. Loree, Eric C. Sayre, Sharlene Gill, Carl J. Brown, Hallie Dau, Mary A. De Vera

**Affiliations:** 1 Faculty of Pharmaceutical Sciences, University of British Columbia, 2405 Wesbrook Mall, Vancouver, BC Canada V6T 1Z3 Canada; 2 Collaboration for Outcomes Research and Evaluation, 2405 Wesbrook Mall, Vancouver, BC Canada V6T 1Z3 Canada; 3 Division of Medical Oncology, Department of Medicine, Faculty of Medicine, University of British Columbia, 2775 Laurel Street, 11th Floor, Vancouver, BC V5Z 1M9 Canada; 4 BC Cancer, 600 W 10th Ave, Vancouver, BC V5Z 4E6 Canada; 5 Arthritis Research Canada, 5591 No 3 Rd, Richmond, BC V6X 2C7 Canada; 6 Department of Surgery, Faculty of Medicine, University of British Columbia, 2775 Laurel Street, 11th Floor, Vancouver, BC V5Z 1M9 Canada; 7 St. Paul’s Hospital, 1081 Burrard St, Vancouver, BC V6Z 1Y6 Canada

**Keywords:** Young-adult cancer, Epidemiology, Incidence, Meta-analysis, Cancer

## Abstract

**Background:**

Recent data suggest that the risk of young-onset colorectal cancer (yCRC), in adults less than 50 years of age, is increasing. To confirm findings and identify contemporary trends worldwide, we conducted a systematic review of studies examining population-level trends in yCRC epidemiology.

**Methods:**

We searched MEDLINE (1946–2018), EMBASE (1974–2018), CINAHL (1982–2018), and Cochrane Database of Systematic Reviews (2005–2018) for studies that used an epidemiologic design, assessed trends in yCRC incidence or prevalence, and published in English. Extracted information included country, age cut-off for yCRC, and reported trends in incidence or prevalence (e.g. annual percent change [APC]). We pooled similarly reported trend estimates using random effects models.

**Results:**

Our search yielded 8695 articles and after applying our inclusion criteria, we identified 40 studies from 12 countries across five continents. One study assessed yCRC prevalence trends reporting an APCp of + 2.6 and + 1.8 among 20–39 and 40–49 year olds, respectively. 39 studies assessed trends in yCRC incidence but with substantial variability in reporting. Meta-analysis of the most commonly reported trend estimate yielded a pooled overall APCi of + 1.33 (95% CI, 0.97 to 1.68; *p* < 0.0001) that is largely driven by findings from North America and Australia. Also contributing to these trends is the increasing risk of rectal cancer as among 14 studies assessing cancer site, nine showed an increased risk of rectal cancer in adults less than 50 years with APCi up to + 4.03 (*p* < 0.001).

**Conclusions:**

Our systematic review highlights increasing yCRC risk in North America and Australia driven by rising rectal cancers in younger adults over the past two decades.

## Background

Colorectal cancer (CRC) is a heterogeneous disease of the colon and rectum predominantly arising from adenomatous polyps or adenomas [1]. The International Agency for Research on Cancer estimated 1.36 million new cases of CRC in 2012 making it the third most common cancer in the world [2].

While CRC has long been considered a disease of older adults [3], recent data suggest an increasing incidence of young-onset CRC (yCRC), which has largely been defined as adults younger than 50 years of age [3–7]. In 2018, the American Cancer Society lowered the recommended age for average-risk adults to initiate screening from 50 to 45 years [8]. In 2019, Liu et al. extracted cancer incidence data from the International Agency for Research on Cancer (IARC) and reported significant increased risk of yCRC for 11 out of 12 countries, with annual percent change in incidence (APCi) ranging from 0.32 (95% confidence interval [CI], 0.01 to 0.64) in Italy to 9.20 (95% CI, 6.85 to 11.59) in Brazil [9]. Identifying whether these incidence trends for yCRC are also reported in peer-reviewed literature is warranted along with examining prevalence trends in order to inform survivorship support and long-term impacts of yCRC. Our objective was to conduct a systematic review of peer-reviewed, observational studies assessing temporal trends in the incidence (risk) and prevalence (burden) of yCRC.

## Methods

### Search strategy

An information scientist searched Medline (1946-), Embase (1974-), and Cochrane Database of Systematic Reviews (2005-) on the Ovid platform, and CINAHL (1982-) and PsycINFO (1880-) on Ebscohost. Database searches were conducted on January 17, 2018, and then updated on December 3, 2018. To ensure comprehensive capture of both articles that may assess CRC with sub-group reporting allowing extrapolation of yCRC and those that specifically assessed yCRC, we combined two separate but complementary searches. First, we used a broad search strategy with concepts of “colorectal cancer”, “prevalence”, and “incidence” to identify articles on CRC across all ages from which data could be extracted for individuals with yCRC. Second, we used a specific strategy where we additionally incorporated concepts of “young age” and “early” to identify articles that specifically examined yCRC. We used both database dependent subject headings (e.g. Medical Subject Headings in Medline) and keywords (Supplementary Table 1). We additionally conducted a hand search of the reference lists of the included studies. The protocol is registered with the PROSPERO international prospective register of systematic reviews (ID: CRD42018082151). The Preferred Reporting Items for Systematic Reviews and Meta-Analysis (PRISMA) was applied to our reporting.

### Study selection

We used the following inclusion criteria: 1) original study using epidemiologic design; 2) published in a peer-reviewed journal as a full-length article or letter; 3) patient population with CRC or yCRC; 4) published in English; and 5) assessed trends in the incidence and/or prevalence yCRC, using regression methods (e.g., joinpoint regression, Poisson regression) and reported corresponding estimates (e.g., annual percent change, rate of change of incidence rate). While yCRC has been largely defined in individuals under 50 years [3–7], this may not be the cut-off used in studies and thus, we considered any cut-off for yCRC. We did not consider grey literature such as annual reports from cancer societies as, in our experience, they may not routinely report on yCRC.

### Data extraction, quality assessment, and meta-analysis

We extracted information on country, data source, sample size, sex distribution, age cut-off for yCRC, and cancer site (e.g. colon, rectum). The primary outcome was measures of trends in the incidence (e.g. APCi) and prevalence (e.g. APCp) of yCRC. As we noted substantial variability in the reporting of trends during data extraction, we contacted authors to request specific estimates (e.g. overall APC) to facilitate pooling. Where available from the included studies, we also extracted reported incidence rates. As some of the studies meeting inclusion criteria additionally reported on outcomes such as yCRC mortality and/or survival, we considered these as secondary outcomes and extracted relevant information. Two researchers (KS and MDV) independently screened titles and abstracts, reviewed manuscripts, and extracted data, resolving any discrepancies by consensus.

We assessed the quality of included studies with a checklist adapted for this systematic review based on the Joanna Briggs Institute Prevalence Critical Appraisal Tool, developed to address the lack of critical appraisal tools for systematic reviews of studies reporting prevalence [10], and the Appraisal tool for Cross-Sectional Studies, developed to address study design, reporting quality and risk of bias in epidemiologic studies of disease prevalence [11]. We selected relevant criteria from each to create a checklist involving 20 items, with each item scored as 1 (“demonstrated in the study”) or 0 (“not demonstrated in study” or “unclear”) (Supplementary Table 2). Item scores were summed with higher scores indicating studies of higher quality.

To synthesize findings on trends in yCRC epidemiology across included studies, we pooled the most commonly reported estimate, in particular, the APCi. We applied methods described by Sheu et al. for meta-analysis using linear mixed method [12] and fit random effects models that assigned within-study variances based on standard errors of APC estimates, with between-study variance estimated by restricted maximum likelihood. It should be noted that none of the included studies reported a standard error with the APCi, however, these were derived using reverse Z-tests on either the reported confidence intervals or, if none were provided, the reported *p*-values. When a p-value was reported only as <X (e.g., < 0.01), we based our computation of the standard error on the conservative assumption that *p* = X. The intercept-only fixed effects solution represents the synthesized common APCi within the group of studies. We obtained pooled estimates of APCi and corresponding 95% confidence intervals (CI) and *p*-values across included studies. We pooled overall APCi and sex-specific APCi, given that studies varied in reporting. We also pooled APCi according to continent. Primarily analysis considered all studies that reported APCi. We also conducted various sensitivity analyses to account for potential overlap between included studies from the United States of America (USA) that used Surveillance, Epidemiology, and End Results Program (SEER) data (e.g., SEER 9, SEER 13). For each group of studies pooled, we computed the Cochran’s Q-test and the I-squared statistic to measure the presence of, and degree of heterogeneity [13]. All analyses were performed using SAS v9.4 (SAS Institute, Cary, North Carolina).

## Results

**Fig. 1:**
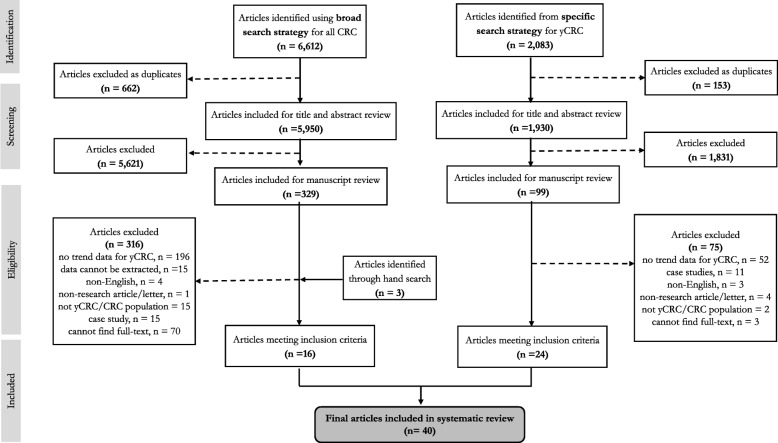
PRISMA flow diagram for systematic review. This figure summarizes systematic review steps, combining the broad and specific search strategies as described in the Methods. Reporting is in accordance to Preferred Reporting for Items for Systematic Review and Meta-Analysis (PRISMA)

The combined search strategies yielded 8695 (6612 with the broad search strategy and 2083 with the specific search strategy) titles (Fig. 1 provides a PRISMA flow diagram). After applying our inclusion criteria, we identified 40 studies – 39 reported trends in yCRC incidence and one in yCRC prevalence. Table 1 summarizes characteristics and quality assessment scores of included studies, according to continent.

**Table 1:** Characteristics of included studies according to continent/region

Study	Country	Population /Data Source	Cancer Information			yCRC age range (yr)	N (yCRC cases)	Outcomes		Quality Score
			Site	Definition	Stage			Primary/ Secondary	Incidence/Prevalence Trend	
**North America (*n*** **= 26 studies)**										
Chow, 1991 [14]	USA	SEER-9	colon	ICD-O	not reported	< 55	not reported	incidence	AAPC x sex x ethnicity	18
Polednak, 1994 [15]	USA	Connecticut Tumor Registry	colorectal	ICD-O	not reported	0 to 44	not reported	incidence	% change in ASR x sex	18
Marrett, 2002 [16]	Canada	National Cancer Incidence Reporting System, CCR	colorectal	ICD-9	not reported	20 to 44	w: 2692 m: 2876	incidence	AAPC x sex	18
Cress, 2006 [17]	USA	SEER-13	colorectal, rectum	ICD-O-3	in situ, invasive, localized, regional/distant	0 to 49	w: 6893 m: 7803	incidence	1. APC x sex 2. APC x sex x site	17
Siegel, 2009 [4]	USA	SEER-13	colorectal	ICD-O-3	local, regional, distal	20 to 49	w: 9733 m: 10, 913	incidence	APC x sex	16
Meyer, 2010 [18]	USA	SEER-9	colon, rectum	not reported	not reported	< 20 to 39	w: 3662 m: 3999	incidence	1. APC x site 2. APC x sex x site	17
Merrill, 2011 [19]	USA	SEER-9	colorectal	ICD-O-2	not reported	30 to 49	not reported	incidence	% change in RAIR x sex x ethnicity	16
Ellison, 2012 [20]	Canada	CCR	colon, rectum	ICD-O-3	not reported	20 to 49	not reported	prevalence	APC x age	16
Giddings, 2012 [21]	USA	California Cancer Registry	colorectal	ICD-O-3	localized, regional, distant	< 50	w: 1278 m: 1259	incidence	APC x sex x ethnicity	19
Nancy You, 2012 [5]	USA	National Cancer Database	colon, rectum	ICD-O-3	stage III, IV	< 50	64,068	incidence	APC x site	18
Austin, 2014 [22]	USA	CDC NPCR	proximal colon, distal colon, rectum	ICD-O-3	local, regional, distal	< 50	not reported	incidence	1. APC x ethnicity 2. APC x sex x ethnicity	18
Siegel, 2014 [23]	USA	SEER-13, CDC NPCR	proximal colon, distal colon, rectum	ICD-O-3	local, regional, distal	< 50	w: 6250 m: 7270	incidence mortality	APC x site	17
Singh, 2014 [24]	USA	California Cancer Registry	proximal colon, distal colon, rectum	ICD-O-3	local, regional, or distant	20 to 49	20,520	incidence	BAPC x sex x age	19
Bailey, 2015 [6]	USA	SEER-9	colon, rectum	not reported	localized, regional, distant	20 to 49	30,708	incidence	1. APC x age 2. APC x age x site	18
Rahman, 2015 [25]	USA	SEER-18, North American Association of Central Cancer Registries	colorectal	not reported	stage 0, I, II, II, IV	< 50	60,023	incidence mortality	AAPC	17
Patel, 2016 [26]	Canada	National Cancer Incidence Reporting System, CCR, Quebec Cancer Registry	colon, rectum	ICD-O-3	not reported	15 to 49	1969: 756 2010: 1475	incidence	1. APC x age 2. APC x sex x age	20
Koblinkski, 2017	US	SEER-18	colorectal	not reported	local, regional, distal	< 50	not reported	incidence	percent change x ethnicity x stage	16
Sheneman, 2017 [27]	US	Colorado Cancer Registry	colorectal	ICD-O-3	early, late	< 50	3729	incidence	1. EAPC 2. EAPC x sex	18
Siegel, 2017 [7]	US	SEER-9, CDC NPCR	proximal colon, distal colon, rectum	ICD-O-3	local, regional, distal	0 to 49	w: 6650 m: 7550	incidence mortality	1. AAPC x site 2. IRR x site	17
Siegel, 2017 [7]	US	SEER-9	proximal colon, distal colon, rectum	ICD-O-3	not reported	20 to 49	not reported	incidence	APC x age x site	19
Wang, 2017 [28]	US	Texas Cancer Registry	colorectal	ICD-O-3	localized, regional, distant	20 to 49	13,028	incidence mortality	APC x age	17
Ansa, 2018 [29]	US	SEER-18	proximal colon, distal colon, rectum	ICD-O-3	localized, regional, distant, or unstaged	0 to 49	57,938	incidence	APC x age	18
Crosbie, 2018 [30]	US	SEER-9	colorectal	ICD-O-3	not reported	20 to 49	w: 4010 m: 4578	incidence	APC x sex	19
Ellis, 2018 [31]	US	California Cancer Registry	colorectal	not reported	in situ, localized, regional, distant	20 to 49	w: 1304 m: 1276	incidence	TAPC x sex x ethnicity	18
Garcia, 2018 [32]	US	SEER-18, CDC NPCR	colorectal	ICD-O-3	localized, regional, distant	20 to 49	not reported	incidence	relative change in IR	18
Jacobs, 2018 [33]	US	SEER-9	colon, rectum	ICD-O-3	Stage 0–2, 3, 4	< 55	not reported	incidence	% change of IR	19
**Oceania (*n*** **= 4 studies)**										
Haggar, 2012 [34]	Australia	Western Australia Data Linkage Service	colorectal	ICD-O-3	not reported	15 to 39	500	incidence mortality	APC x sex	18
Boyce, 2016 [35]	Australia	New South Wales Central Cancer Registry	colon, rectum	ICD-O-3 and ICD-10	localised, regional, distant	< 30 to 49	w: 971 m: 1030	incidence mortality	average annual linear trend in R	19
Gandhi, 2017 [36]	New Zealand	New Zealand Cancer Registry	proximal colon, distal colon, rectum	not reported	not reported	< 50	not reported	incidence	rate of change of IR	19
Troeung, 2017 [37]	Australia	Western Australia Cancer Registry	colorectal	ICD-9 and ICD-10	tumour grade	15 to 39	w: 256 m: 261	incidence mortality	1. APC overall 2. APC x sex	19
**Europe (*n*** **= 3 studies)**										
Zaridze, 1990 [38]	Russia	not well described	colon, rectum	not reported	not reported	<29 to 49	not reported	incidence	APC x type x sex x age	9
Larsen, 2010 [39]	Norway	Cancer Registry of Norway	colon, rectum	ICD-7	not reported	35 to 54	w: 1739 m: 1707	incidence	APC x age	18
Ullah, 2018 [40]	Ireland	National Cancer Registry of Ireland	colorectal	not reported	not reported	20 to 49	2750	incidence	APC x age	18
**Asia (*n*** **= 6 studies)**										
Nooyi, 2011 [41]	India	Indian Population-Based Cancer Registries	rectum	ICD-O	not reported	35 to 49	not reported	incidence	EAPC x sex x age	16
Wu, 2012 [42]	China	Shanghai Cancer Registry	colorectal	ICD-9	not reported	15 to 49	w: 312 m: 259	incidence	APC x sex	19
Zhou, 2015 [43]	China	Guangzhou Cancer Registry	colon, rectum	ICD-10	not reported	< 50	not reported	incidence	1. APC 2. APC x sex	18
Nakagawa, 2017 [44]	Japan	Japanese Population-Based Cancer Registries	colon, rectum	ICD-10	not reported	< 50	not reported	incidence	1. APC x overall 2. APC x site	19
Sarakarn, 2017 [45]	Thailand	Khon Kaen Cancer Registry	colorectal	ICD-O	stage I, II, III, and IV	< 50	w: 1566 m: 1798	incidence	1. APC 2. APC x sex	17
Zhang, 2018 [46]	China	Hong Kong Cancer Registry	colon, rectum	not reported	not reported	20 to 49	8829	incidence	APC x sex x type	20
**Africa (*n*** **= 1 study)**										
Hamdi Cherif, 2014 [47]	Algeria	Population-Based Cancer Registry of Setif	colorectal	ICD-O-3	not reported	15 to 44	not reported	incidence	APC x sex	19

Abbreviations: *APC* annual percent change (in incidence or prevalence); *AAPC* average annual percent change; *ASR* age-standardized incidence rate; *BAPC* biannual annual percent change; *EAPC* estimated annual percent change; *TAPC* triannual percent change; *IR* incidence rate; *w* women; *m* men; *yCRC* young-onset colorectal cancer; *CDC* Centre for Disease Control; *ICD-O* International Classification of Diseases for Oncology; *ICD* International Classification of Diseases;

*SEER* Surveillance, Epidemiology, and End Results Program Registry;

*CCR* Canadian Cancer Registry;

*CDC NPCR* Centre for Disease Control National Program for Cancer Registries

### Trends yCRC prevalence

A 2012 Canadian study by Ellison et al. evaluating trends in the prevalence of various cancers reported APCps of yCRC of + 2.6 (*p* < 0.01) among 20–39 year-olds and + 1.8 (*p* < 0.01) among 40–49 year-olds, suggesting an increasing burden over the study period of 2002 to 2008 [20].

### Trends in yCRC incidence

Trends in the incidence of yCRC were reported in 39 studies, with 31 published after 2010 including seven published in the past year (2018) alone [29–33, 40, 46]. Altogether, 31 studies defined yCRC based on a cut-off of diagnosis below the age of 50 years, two based on 40 years [18, 34], three based on 45 years [15, 16, 47], and three based on 55 years [14, 33, 39]. Incidence rates for yCRC were reported in 17 studies [14, 15, 17, 18, 21, 28, 29, 31, 32, 35–38, 40, 42, 48] (Supplementary Table 3). We estimated a pooled incidence rate per 100,000 for yCRC of 8.0 (95% confidence interval [CI], 5.8 to 10.3) from seven studies reporting overall rates similarly (e.g. no additional sub-groups such as age) from 1982 to 2014 [17, 21, 30, 32, 35, 37, 48] (Supplementary Figure 1). We estimated pooled incidence rates per 100,000 for yCRC of 6.4 (95% CI, 4.0 to 8.7) among women (Supplementary Figure 2) and 6.8 (95% CI, 3.6 to 10.1) among men (Supplementary Figure 3) from 1982 to 2014 [15, 17, 21, 30, 37, 42].

With respect to our primary outcome of trends in yCRC incidence, included studies varied across reported trend measures – for example APCi in 22 studies [4, 5, 17, 18, 21–23, 26, 28–30, 34, 37, 38, 40, 42–44, 46, 47, 49, 50], extensions of the APCi (average APCi [AAPCi], estimated APCi [EAPCi]) in 10 studies [14, 16, 24, 25, 27, 31, 39, 41, 45, 48], and other measures such as % changes in incidence rates, incidence rate ratios, and odds ratios in seven studies [15, 19, 32, 33, 35, 36, 51]. Studies also varied in how they reported these incidence trends – 15 provided overall estimates [5, 6, 18, 23, 25, 28, 29, 32, 33, 35, 40, 44, 48, 50, 51], 17 according to sex [4, 14–17, 19, 21, 24, 31, 34, 36, 38, 39, 41, 42, 46, 47], and seven provided both overall and sex-specific estimates [22, 26, 27, 30, 37, 43, 45].

Table 2 summarizes results of meta-analyses of the most commonly reported trend estimate - the APCi – overall, by sex, and by continent. Overall, we obtained pooled APCi of + 1.33 (95% CI, 0.97 to 1.68; *p* < 0.0001). When we meta-analyzed studies that reported sex-specific APCi, we obtained a pooled APCi of + 1.02 (95% CI, 0.21 to 1.83; *p* = 0.02) for women and + 0.99 (95% CI, 0.31 to 1.67; *p* = 0.006) for men. Further columns in Table 2 show no marked changes in pooled APCi overall with various sensitivity analyses that attempted to account for potential overlap in data from included studies from the USA, particularly those using SEER. In the following, we present our synthesis of findings on trends in yCRC incidence according to continent with Table 3 summarizing reported trends.

**Table 2:**
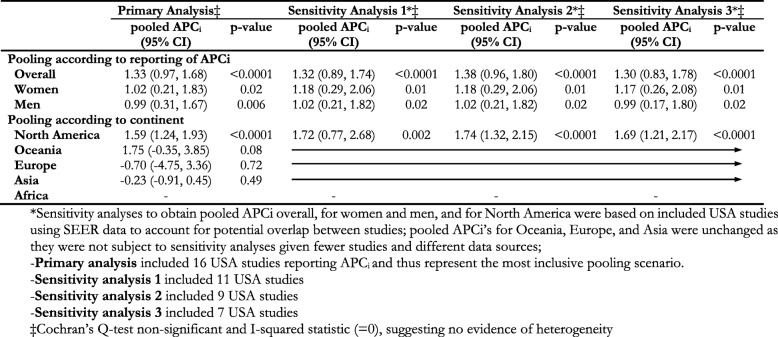
Pooled annual percent change in incidence (APC_i_), 95% confidence intervals, and p-values for yCRC overall, according to sex, and continent

*Sensitivity analyses to obtain pooled APCi overall, for women and men, and for North America were based on included USA studies using SEER data to account for potential overlap between studies; pooled APCi’s for Oceania, Europe, and Asia were unchanged as they were not subject to sensitivity analyses given fewer studies and different data sources;

-**Primary analysis** included 16 USA studies reporting APC_i_ and thus represent the most inclusive pooling scenario.

-**Sensitivity analysis 1** included 11 USA studies

-**Sensitivity analysis 2** included 9 USA studies

-**Sensitivity analysis 3** included 7 USA studies

‡Cochran’s Q-test non-significant and I-squared statistic (=0), suggesting no evidence of heterogeneity

**Table 3:** Reported trends in incidence of yCRC incidence overall and according to sex

Study	Date Range	Overall	Women	Men	Findingb
**North America**					
Chow 1991 [1991]	1976–1987	–	CC AAPC White: − 2.0 (< 0.05) CC AAPC Black: − 1.3	CC AAPC White: − 0.7 CC AAPC Black: + 1.7	not consistent
Polednak, 1994 [15]	1965–1991	–	CRC % change ASR: − 19 (*p* = 0.153)	CRC % change ASR: − 29 (*p* < 0.05)	↓ CRC m
Marrett 2002 [16]	1969–1996	–	CRC AAPC: − 1.39 (− 1.69, − 1.08)	CRC AAPC: − 0.43 (− 0.77, − 0.08)	↓ CRC w ↓ CRC f
Cress, 2006 [17]	1992–2001	CRC APC: + 1.1^a^	CRC APC: + 1.4 RC APC: + 3.6 (*p* < 0.05)	CRC APC: + 0.8 RC APC: + 2.5 (*p* < 0.05)	↑ RC w ↑ RC m
Siegel 2009 [4]	1992–2005	–	CRC APC: + 1.6 (*p* < 0.05)	CRC APC: + 1.5 (*p* < 0.05)	↑ CRC w ↑ CRC m
Meyer 2010 [18]	1973–2005	CC APC: − 0.2 (− 0.6, 0.3) RC APC: + 2.6 (1.9, 3.3)	RC APC: + 2.5 (1.8, 3.8)	RC APC: + 2.5 (1.6, 3.4)	↑ RC
Merrill, 2011 [19]	2000–2007	–	CRC % change RAIR White: 21.7 CRC % change RAIR Black: 11.4	% change CRC RAIR White: 2.0 CRC % change RAIR Black: 0.4	↑ CRC w ↑ CRC m
Giddings, 2012 [21]	1998–2007	–	CRC APC Chinese: −1.8 (− 3.9, 0.3) CRC APC Japanese: − 0.1 (− 3.6, 3.7) CRC APC Filipino: − 0.1 (− 2.2, 2.1) CRC APC Korean: + 0.5 (− 2.0, 3.1) CRC APC South Asian: - CRC APC Vietnamese: + 2.2 (− 0.8, 5.2)	CRC APC Chinese: − 1.6 (− 3.3, 0.1) CRC APC Japanese: + 1.4 (− 2.5, 5.6) CRC APC Filipino: + 0.6 (− 1.6, 2.9) CRC APC Korean: + 3.4 (0.1, 6.7) CRC APC South Asian: + 1.5 (− 2.9, 6.2) CRC APC Vietnamese: + 1.8 (− 0.8, 4.4)	not consistent
Nancy You 2012 [5]	1998–2007	CRC APC: + 2.1 (1.1, 3.1) CC APC: + 2.7 (2.0, 3.3) RC APC: + 3.9 (3.1, 4.7)	–	–	↑ CRC ↑ CC ↑ RC
Austin 2014 [22]	1998–2009	CRC APC N Hispanic White: + 1.69 (1.47, 1.91) Black: + 0.44 (− 0.03, 0.92) Asian: + 0.61 (− 0.41, 1.35) Hispanic White: + 0.59 (− 0.15, 1.33)	CRC APC N Hispanic White: + 1.79 (1.46, 2.11) Blacks: + 0.47 (− 0.39, 1.34) Asian: + 0.45 (− 0.57, 1.49) Hispanic White: + 0.76 (0.03, 1.5)	CRC APC N Hispanic White: + 1.61 (1.35, 1.87) Blacks: + 0.40 (− 0.14, 0.93) Asian: + 0.72 (− 0.53, 1.99) Hispanic White: + 0.42 (− 0.63, 1.48)	not consistent
Siegel 2014 [23]	2001–2010	CRC APC: + 1.1 (*p* < 0.05) RC APC: + 1.8 (*p* < 0.05)	–	–	↑ CRC ↑ RC
Singh, 2014 [23]	1988–2009	–	CRC BAPC 20-29y: + 3.8 (*p* < 0.011) CRC BAPC 30-39y: + 4.5 (*p* < 0.001) CRC BAPC 40-49y: + 2.6 (*p* < 0.001)	CRC BAPC 20-29y: + 2.7 (*p* < 0.011) CRC BAPC 30-39y: + 3.5 (*p* < 0.001) CRC BAPC 40-49y: + 2.7 (*p* < 0.001)	↑ CRC w ↑ CRC m
Bailey 2015 [49]	1975–2010	CRC APC 20-34y: + 1.99 (1.48, 2.51) CRC APC 35-49y: + 0.41 (0.14, 0.69) RC APC 20-34y localized: + 4.03 (*p* < 0.001) regional: + 3.05 (*p* < 0.001) distant: + 2.66 (*p* < 0.001) RC APC 35-49y localized: + 1.62 (*p* < 0.001) regional: + 1.37 (*p* < 0.001) distant: + 1.46 (*p* < 0.001)	–	–	↑ CRC ↑ RC
Rahman 2015 [25]	1992–2009	CRC AAPC: + 1.68 (*p* < 0.05)	–	–	↑ CRC
Patel 2016 [26]	1997 to 2010	CRC APC 15-29y: + 6.7 (4.3, 9.3) CRC APC 30-39y: + 2.4 (1.5, 3.3) CRC APC 40-49y: + 0.8 (0.3, 1.4)	CRC APC 15-29y: + 7.9 (1.1, 15.1) CRC APC 30-39y: + 2.3 (0.8, 3.7) CRC APC 40-49y: + 0.6 (0.1, 1.2)	CRC APC 15-29y: + 7.0 (3.7, 10.4) CRC APC 30-39y: + 2.5 (1.5, 3.4) CRC APC 40-49y: + 1.0 (0.4, 1.5)	↑ CRC
Koblinski 2017 [51]	2000–2010	CRC % change Hispanic localized:↑77%; regional: ↑56% distant: ↑57% CRC % change White localized:↑21%; regional: ↑18% distant: ↑41%	–	–	↑ CRC
Sheneman 2017 [27]	2003–2013	CRC EAPC: + 1.7	CRC EAPC: + 0.6	CRC EAPC: + 2.7 (*p* < 0.05)	↑ CRC m
Siegel 2017 [7]	2004–2013	CRC AAPC: + 1.6 (*p* < 0.05) CRC IRR: + 1.22 (1.17, 1.28) RC AAPC: + 2.0 (*p* < 0.05) RC IRR: + 1.22 (1.13, 1.31)	–	–	↑ CRC ↑ RC
Siegel 2017 [7]	1974–2013	CC APC 20-29y: + 2.4 (1.6, 3.3) CC APC 30-39y: + 1.0 (0.5, 1.5) CC APC 40-49y: + 1.3 (0.7, 1.8) RC APC 20-29y: + 3.2 (2.4, 3.9) RC APC 30-39y: + 3.2 (2.7, 3.7) RC APC 40-49y: + 2.3 (1.8, 2.7)	–	–	↑ CC ↑ RC
Wang 2017 [28]	1995–2010	CRC APC 20-39y: + 1.82 (*p* < 0.01) CRC APC 40-49y: + 0.33 (*p* = 0.08)	–	–	no consistent
Ansa, 2018 [29]	2000–2014	CRC APC < 40: + 2.7 (*p* < 0.001) CRC APC 40-49y: + 1.7 (*p* < 0.001)	–	–	↑ CRC
Crosbie, 2018 [30]	1992–2014	CRC APC: + 1.8 (*p* < 0.05)	CRC APC: + 1.8 (*p* < 0.05)	CRC APC: + 1.7 (*p* < 0.05)	↑ CRC
Ellis, 2018 [31]	2010–2014	–	CRC TAPC Chinese: + 0.1 (− 2.1, 2.4) CRC TAPC Japanese: + 0.5 (− 3.1, 4.1) CRC TAPC Filipino: − 0.6 (− 3.5, 2.4) CRC TAPC Korean: + 0.8 (− 3.8, 5.5) CRC TAPC South Asian: + 4.3 (− 2.0, 10.9) CRC TAPC Vietnamese: − 0.5 (− 3.1. 2.2) CRC TAPC SEast Asian: - CRC TAPC White: + 1.9 (0.8, 2.9) CRC TAPC Black: + 0.3 (− 0.7, 1.4) CRC TAPC Hispanic: + 2.1 (1.2, 3.1)	CRC TAPC Chinese: + 0.4 (− 2.0, 2.9) CRC TAPC Japanese: + 1.5 (− 2.0, 5.0) CRC TAPC Filipino: + 1.1 (− 1.1, 3.3) CRC TAPC Korean: + 0.7 (− 1.8, 3.3) CRC TAPC South Asian: − 0.9 (− 5.7, 4.2) CRC TAPC Vietnamese: + 1.1 (− 3.9, 6.2) CRC TAPC SEast Asian: − 1.0 (− 3.8, 1.9) CRC TAPC White: + 0.9 (0.4, 1.4) CRC TAPC Black: − 0.9 (− 2.2, 0.4) CRC TAPC Hispanic: + 1.6 (0.3, 2.9)	not consistent
Garcia, 2018 [32]	2001–2014	CRC relative increase IR: 24%	–	–	↑ CRC
Jacobs, 2018 [33]	1973–2014	CC % change IR: 41.5 (37.4, 45.8) RC % change IR: 9.8 (6.2, 13.6)			↑ CRC ↑ RC
**Oceania**					
Haggar 2012 [34]	1982–2007	–	CRC APC: + 1.4 (0.1, 2.7)	CRC APC: − 0.4 (− 1.7, 1.0)	↑CRC w
Boyce 2016 [35]	2001–2008	CRC OR: 1.03 (0.99, 1.07)	–	–	no change
Gandhi 2017 [36]	1975–2012	–	RC IRR: 1.13 (1.2, 1.26)	RC IRR: 1.18 (1.06, 1.32)	↑ RC w ↑ RC m
Troeung 2017 [37]	1982–2007	CRC APC: + 3.0 (0.7, 5.5)	CRC APC: + 3.4 (1.1, 5.7)	CRC APC: + 2.6 (− 0.9, 5.2)	↑ CRC w
**Europe**					
Zaridze, 1990 [38]	1971–1987	–	CC APC <29y: − 0.1 (− 14.2, 14.3) CC APC 30-39y: − 1.3 (− 7.4, 5.1) CC APC 40-49y: + 8.2 (4.6, 11.9) RC APC < 29y: − 13.7 (− 26.4, 0.2) RC APC 30-39y: − 9.1 (− 18.3, 1.2) RC APC 40-49y: + 4.3 (0.5, 8.3)	CC APC <29y: − 9.1 (− 17.2, − 0.3) CC APC 30-39y: − 2.9 (− 9.7, 4.5) CC APC 40-49y: + 3.2 (− 0.1, 6.6) RC APC < 29y: − 16.5 (− 29.3, − 1.5) RC APC 30-39y: − 11.1 (− 16.4, − 5.4) RC APC 40-49y: + 3.7 (− 1.4, 9.1)	no consistent
Larsen 2010 [39]	1992–2006	–	proximal CC EAPC: ≥ − 2 distal CC EAPC: − 1 RC EAPC: <+ 1	proximal CC EAPC: < 1 distal CC EAPC: ≥ − 2 RC EAPC: <+ 1	no change w no change m
Ullah, 2018 [40]	1994–2012	CRC APC 20-29y: + 9.17 (*p* < 0.03) CRC APC 30-39y: + 4.6 (*p* = 0.1) CRC APC 40-49y: + 0.83 (*p* = 0.45)	–	–	not consistent
**Asia**					
Nooyi, 2011 [41]	1983–2002	–	RC EAPC 35–39 y: - RC EAPC 40-44y: + 1.7 (*p* = 0.35) RC EAPC 45–49y: + 0.4 (*p* = 0.83)	RC EAPC 35-39y: + 3.1 (*p* = 0.12) RC EAPC 40-44y: + 1.8 (*p* = 0.29) RC EAPC 45–49y: + 1.4 (*p* = 0.41)	no change w no change m
Wu 2012 [42]	1973–2005	–	CRC APC: − 0.3 (− 0.9, 0.3)	CRC APC: 0.1 (− 0.4, 0.4)	no change w no change m
Zhou 2015 [43]	2005–2011	CRC APC: −3.07 (*p* < 0.01)	CRC APC: −2.56 (*p* = 0.21)	CRC APC: −3.45 (*p* = 0.06)	↓ CRC
Nakagawa 2017 [44]	1987–2004	CRC APC: −0.8 (−1.7, 0.1) RC APC: − 1.9 (− 2.6, − 1.1)	–	–	↓ RC
Sarakarn 2017 [45]	1989–2012	CRC AAPC: + 5.7	CRC AAPC: + 5.7 (*p* < 0.05)	CRC AAPC: + 3.2 (*p* < 0.05)	↑ CRC w ↑ CRC m
Zhang, 2018 [46]	1983–2012		CC APC: − 1.56 (− 1.73, − 1.39) RC APC: − 0.17 (− 0.40, 0.05)	CC APC: − 1.11 (− 1.32, 0.90) RC APC: + 0.60 (0.37, 0.84)	not consistent
**Africa**					
Hamdi Cherif, 2014 [47]	1986–2010	–	CRC APC: − 2.1 (− 6.3, 2.3)	CRC APC: − 0.8 (− 4.7, 3.3)	no change w no change m

^a^- obtained from authors after contacting them; ^b^-key finding(s) indicate consistent trends identified from each study

Abbreviations: *CRC* colorectal cancer; *RC* rectal cancer; *CC* colon cancer; *APC* annual percent change; *AAPC* average annual percent change; *ASR* age-standardized incidence rates; *EAPC* estimated annual percent change; *RAIR* risk-adjusted incidence rate; *BAPC* biannual percent change; *TAPC* triannual percent change; *IRR* incidence rate ratio; *OR* odds ra

#### North America

The majority of studies in our systematic review are from North America with 25 studies altogether [4–7, 14–30, 32, 33, 48, 51] and 23 from the USA [4–7, 14, 15, 17–19, 21–25, 27–30, 32, 33, 48, 51]. Among studies from the USA, 12 reported overall estimates and consistently showed increasing incidence of yCRC, largely driven by rectal cancer in eight studies [5, 17, 18, 23, 33, 48, 50]. The earliest of these studies by Meyer et al. in 2010 analyzed SEER-9 data and reported an APCi of + 2.6 (95% CI, 1.9 to 3.3) for rectal cancer and − 0.2 (95% CI, − 0.6 to 0.3) for colon cancer [18]. Using SEER-9 data in 2015, Bailey et al. highlighted the increasing risk of rectal cancer, with APCi’s of + 4.03 (*p* < 0.001) for localized, + 3.05 (*p* < 0.001) for regional, and + 2.66 (*p* < 0.001) for distant disease and estimated that incidence rates of rectal cancers for patients under 50 years are expected to increase up to 124.2% by 2030 [6]. Siegel et al. published one study in 2009 (SEER-13) [4], one in 2014 (SEER-13) [23], and two in 2017 (SEER-9) [7, 48] that consistently showed the contributions of rectal cancer to the increasing risk of yCRC. In their most recent study in 2017, they showed that the age-adjusted proportion of incident cases in adults 55 years and younger increased from 14.6% (95% CI, 14.0 to 15.2%) to 29.2% (95% CI, 28.5 to 29.9%) for rectal cancer (18). Of note, studies from the USA also allowed for evaluation of sex-specific and ethnicity-specific trends in yCRC incidence. Eight studies reported estimates according to sex with four showing increasing incidence of yCRC in both women and men [4, 17, 19, 24]. Ethnicity-specific trends were reported in 17 USA studies [4, 5, 14, 18, 19, 21, 22, 24, 25, 27–33, 48, 51]. We observed consistently reported increases in yCRC incidence among non-Hispanic White [4, 22, 48], White [18, 19, 51], and Black patients [18].

We also identified two studies from Canada [16, 20, 26]. In 2002, Marrett et al. reported decreasing incidence of yCRC with AAPCi’s from 1969 to 1996 of − 1.39 (95% CI, − 1.69 to − 1.08) for women between 20 and 44 years and − 0.43 (95% CI, − 0.77 to − 0.08) for men [16]. However, the more recent study in 2016 by Patel et al. reported APCi values ranging from + 0.6 (95% CI, 0.1 to 1.2; 40 to 49 years) to + 7.9 (95% CI, 1.1 to 15.1; 15 to 29 years) for women and from + 1.0 (95% CI, 0.4 to 1.5; 40 to 49 years) to + 7.0 (95% CI, 3.7 to 10.4; 15 to 29 years) for men [26].

Altogether, when we meta-analyzed APCi’s reported overall across studies from North America, we obtained a pooled APCi of + 1.59 (95% CI, 1.24 to 1.93; *p* < 0.0001). Various pooling scenarios that attempted to account for potential overlap in data from included USA studies did not result in marked changes, though somewhat higher pooled APCi’s (as shown in Table 2) suggest that meta-analyzing across all included studies yielded the most conservative (e.g. lowest) estimate.

#### Oceania

We included four studies from Oceania [34–37]. Three studies from Australia showed an increasing risk of yCRC [34, 35, 37], particularly among women [34, 37]. In 2012, Haggar et al. showed this increasing trend in yCRC among women (APCi, + 1.4; 95%, 0.1 to 2.7) but not for men (APCi, − 0.4; 95%, − 1.7 to 1.0) [34]. In 2017, Troeung et al. similarly found increasing risk of yCRC among women (APCi, + 3.4; 95% CI, 1.1 to 5.7) but not among men (APCi, + 2.6; 95% CI, − 0.9 to 5.2) [37]. In 2017 in New Zealand, Gandhi et al. reported incidence rate ratios that suggested increased risk of rectal cancer for both women (IRR 1.13; 95% CI, 1.2 to 1.26) and men (IRR 1.18; 95% CI, 1.06 to 1.32) less than 50 years [36]. Meta-analyzing reported overall APCi’s yielded the highest point estimate across all continents of 1.75 for Oceania, though not statistically significant (95% CI, − 0.35 to 3.85; *p* = 0.08).

#### Europe

We included three studies from Europe [38–40]. In a Russian study, Zaridze et al. reported APCi’s according to sex, cancer type, and age group but no consistent trends in yCRC epidemiology were noted [38]. In their 2010 study, Larsen and Bray did not show significant changes in yCRC incidence among 35 to 54 year-olds, with an APCi between − 2 and + 1 for both women and men [39]. The most recent study in 2018 from Ireland by Ullah et al. reported inconsistent findings with APCi of + 9.17 (*p* < 0.03) for 20 to 29 year-olds, + 4.6 (*p* = 0.1) for 30 to 39 year-olds, and + 0.83 (*p* = 0.45) for 40 to 49 year-olds [40]. When we meta-analyzed reported overall APCi’s, we obtained a pooled APCi of − 0.70 (95% CI, − 4.76 to 3.36; *p* = 0.72) for European studies.

#### Asia

We identified six studies from Asia [41–46]. The only increasing trend for yCRC was found in Thailand by Sarakarn et al. who reported an AAPCi of + 5.7 between 1989 and 2012 for patients under 50 years overall, and significant trends for both women (AAPCi, + 5.7; *p* < 0.05) and men (AAPCi, + 3.2; *p* < 0.05) [45]. In contrast, a decreasing trend for rectal cancer in patients less than 50 years was reported in Japan (APCi, − 1.9; 95% CI, − 2.6 to − 1.1) [44]. Three studies from China reported conflicting findings [42, 43, 46]. Zhou et al. (2015) reported a decrease in incidence with an APCi of − 3.1 (*p* < 0.05) for yCRC [43]; Wu et al. reported no change in incidence with APCi’s of − 0.3 (95% CI, − 0.9 to 0.3) in women and + 0.1 (95% CI, − 0.4 to 0.4) in men aged 15 to 49 years [42]; while Zhang et al. did not show consistent findings [46]. When we meta-analyzed similarly reported overall APCi’s, we obtained a pooled estimate of − 0.22 (95% CI, − 0.91 to 0.45; *p* = 0.49) for studies from Asia.

#### Africa

One included study from Africa used the Cancer Registry of Setif, Algeria from 1986 to 2010 and reported no change in yCRC incidence with APCi’s of CRC among patients 15 to 44 years of − 2.1 (95% CI, − 6.3 to 2.3) for women and − 0.8 (95% CI, − 4.7 to 3.3) for men [47].

### Secondary outcomes

Among included studies, seven reported additional information on survival [25, 28, 35] or mortality [23, 34, 37, 48] in yCRC. With respect to survival, Rahman et al. (2015) reported five-year relative survival for yCRC in the USA for Non-Hispanic Whites as 65.5%, African Americans as 56.4%, Hispanics as 62.0%, Asians as 65.9%, and Pacific Islanders, American Indians, and Alaska Natives as 59.8% [25]. In 2017, Wang et al. examined yCRC among Hispanics in the USA and reported a five-year survival proportion of 62.4% among 20 to 39 year-olds and 63.9% among 40 to 49 year-olds [28]. In Australia, Boyce et al. showed that the five-year survival was higher in those with yCRC (< 50 years) as compared to those with average-onset colorectal cancer (aCRC) (≥50 years) (67.1%; 95% CI, 64.5 to 69.6% versus 55.8%; 95% CI, 55.0 to 56.4%, *p* < 0.001) and, compared to patients with aCRC, those with yCRC had a 33% lower risk of disease-related death (adjusted hazard ratio [aHR], 0.67; 95% CI, 0.61 to 0.74) [35]. With respect to trends in yCRC mortality, Haggar et al. reported APCs in age-adjusted mortality rates (per 100,000) from 1982 to 2005 in Western Australia of − 2.3 (95% CI, − 3.7 to − 0.8) among women and − 2.1 (95% CI, − 4.0 to − 0.1) among men [34]. However, in the USA, Siegel et al. reported a 13% increase in mortality rates for yCRC patients from 2000 to 2014 [48].

## Discussion

We identified 40 studies spanning 12 countries across five continents evaluating temporal trends in the incidence and prevalence of yCRC. Altogether, we found an increasing incidence of yCRC with a worldwide pooled APCi of + 1.33 (95% CI, 0.97 to 1.68; *p* < 0.0001), that is largely driven by increasing incidence in the USA, Australia, and Canada with reported overall APCi’s up to + 7.9 (95% CI, 1.1 to 15.1) [26] and nearly 30% increased incidence over 20 years [4, 6]. With comparatively fewer included studies and inconsistent findings, similar conclusions may not necessarily be drawn for studies from Europe, Asia, and Africa. Another finding from our systematic review is that trends of the increasing risk of yCRC appear to be driven by increased rectal cancers shown in nine out of 14 [5, 6, 17, 18, 23, 24, 33, 36, 50] studies that specifically evaluated it and with APCi’s up to + 4.03 (*p* < 0.001) [6].

To our knowledge, this is the first systematic review assessing the changing epidemiology of yCRC. While narrative reviews of yCRC have been published [1, 52, 53], the only prior systematic review specific to yCRC was by O’Connell et al. in 2004, which included 55 studies based on clinical samples of patients [54]. Altogether, studies in this prior systematic review contributed 6425 patients allowing authors to describe clinical characteristics of yCRC including common presenting symptoms (abdominal pain and rectal bleeding), observed delays in diagnosis exceeding six months, and treatment patterns [54]. A specific finding from O’Connell et al.’s prior systematic review that the rectum and sigmoid colon were the most frequent sites (54% of tumours) is consistent with our findings on the contribution of rectal cancer to the increased incidence of yCRC at the population level. Interestingly, the authors found no difference in the sex distribution of yCRC with 48.6% in women and 51.4% in men [54] and our pooled sex-specific APCi’s for women (+ 1.02; 95% CI, 0.20 to 1.83) and men (+ 0.99; 95% CI, 0.31 to 1.67) were similar. These findings have implications for efforts in raising awareness for both women and men on the increasing risk of yCRC, considering biological differences between sexes as well as gender differences, for example, healthcare seeking.

The increasing incidence of yCRC across a number of jurisdictions seen in our systematic review may indeed signal a recent paradigm shift in CRC. Of note, the majority of included studies (*n* = 31) have been published since 2010 with seven published in the past year (2018) alone. Synthesizing these published, peer-reviewed evidence including quality assessment and where feasible, pooling of commonly reported estimates brings areas for attention based on key findings. One of our key findings is the contribution of findings from the USA, Australia, and Canada to the worldwide increased risk of yCRC. Quite timely, our systematic review also builds on Liu et al.’s recently published work taking data from the IARC and using joinpoint regression to calculate APCi’s to show the increasing incidence of yCRC in 11 out of 12 countries [9]. Indeed, it is important to consider these as complementary works as some countries/continents represented in included studies in our systematic review were not captured in the aforementioned study and vice versa.

Along with key findings, it is also important to discuss knowledge gaps that we identified from our systematic review. Notably, only one included study evaluated trends in the prevalence of yCRC [20]. It is important to understand the burden of yCRC in terms of trends in prevalence as it is only through population-level examinations of the number of people who have been previously diagnosed with yCRC that we can count and characterize survivors and ultimately inform the long-term impacts of yCRC. Understanding trends in survival and mortality in yCRC is another area identified in our systematic review requiring further investigation to inform contemporary knowledge of this disease. While we noted comparable five-year survival rates among two USA studies [25, 28], a comparison of mortality data suggests conflicting evidence. Specifically, in terms of mortality trends in yCRC, an Australian study indicated that it has decreased for both sexes from 1982 to 2005 in Western Australia [34] while Siegel et al. reported that it has increased from 2000 to 2014 in the USA [5]. Subsequent to the latter article, the same authors published a letter reporting an increasing mortality trend for yCRC in patients 20 to 54 years from 1970 to 2014 (APC_mortality_*,+* 1.0; 95% CI, 0.7 to 1.4) [50].

Aside from identifying gaps, our systematic review also has implications for informing future research needs for better understanding of yCRC. Indeed, key findings of an increasing risk of yCRC highlights the need for future research examining reasons for this. As well, findings from our systematic review suggesting that the increased risk in yCRC may be driven by increased risk of rectal cancer among younger adults underscores the need for research on etiologic reasons for the differences in the risk between rectal and colon cancers. Among included studies in the systematic review, only one evaluated population-level determinants of yCRC [5]. Specifically, in addition to examining trends in the incidence of yCRC in the USA, You et al. also reported independent determinants or risk factors for advance stage yCRC (Stage III/IV) which included: **1)** younger age (aHR for 18 to 39 year-olds: 1.4, 95% CI, 1.2 to 1.6; aHR for 30 to 39 year-olds: 1.21, 95% CI, 1.1 to 1.4, compared to 40 to 49 year-olds); **2)** African-American ethnicity (aHR, 1.2, 95% CI, 1.1 to 1.3, compared to White); and **3)** insurance status (compared to those with insurance, aHR for those without insurance was 1.2, 95% CI, 1.1 to 1.3; and for those on Medicaid, 1.6, 95% CI, 1.5 to 1.8) [5]. There was no specific evaluation of risk factors with respect to colon or rectal cancer among younger adults. Although they did not evaluate direct associations with yCRC, another included study, by Patel et al. from Canada, evaluated trends in lifestyle factors among Canadians less than 50 years to elucidate if there may be parallel increases with yCRC incidence trends. With a parallel increase in the prevalence of being overweight or obese in adults younger than 50 years, authors described obesity as a potential lifestyle factor influencing the increasing risk of yCRC in Canada [26], which may be consistent in similar countries. Aside from lifestyle factors, there may also be psychosocial factors. For example, the increasing risk of yCRC may be associated with a delay in seeking medical care from young adults [55]. Noteworthy, the observed risk of yCRC may be an under-representation of the true risk due to clinicians dismissing symptoms that may be consistent but not be specific to CRC [5].

Along with providing further confirmatory evidence on the increasing risk of yCRC, findings of our systematic review also lend to the question of the appropriate age for screening for CRC. In 2018, the American Cancer Society lowered the recommended age for average-risk adults to initiate screening from 50 to 45 years [8]. This has been subject to debate with arguments for including evidence for increasing risk of yCRC and expected benefits while arguments against including lower absolute risk of yCRC compared to CRC and potential issues with adherence [56, 57]. Recently in January 2018, Abualkhair et al. analyzed the incidence of CRC in 1-year age increments using SEER-18 data and showed a steep increase in the incidence of invasive CRC from 49 to 50 years (46.1% increase per 100,000) with an IRR of 1.46 (95% CI, 1.42 to 1.51) [58]. Their findings provide support for earlier screening for CRC by showing a substantial burden of undetected cancers among younger adults who would not normally undergo screening at 50 years [58]. Of note, in addition to discussions on the recommended age for screening, it is also important to consider efforts to increase awareness of the increasing risk of yCRC among patients and healthcare providers. In our recent patient-oriented qualitative study on the experiences of individuals with diagnosis and treatment with yCRC, a key finding was a theme on misdiagnosis and/or delays in diagnoses, with some participants sharing that symptoms and concerns were dismissed [59]. This finding is supported by those from the Colorectal Cancer Alliance’s annual survey, which in 2018 reported that 67% of yCRC patients saw at least two physicians before being diagnosed correctly [59, 60].

Strengths and limitations of our systematic review deserve discussion. All database searches were conducted by an experienced information scientist. Combining two separate but complementary searches was a unique feature of our study that maximized our capture of eligible studies. However, the inclusion of relevant studies may have been limited by publication bias as in any other systematic review. We did not consider abstracts given the importance of being able to assess the quality of the included studies. In addition, although we considered articles from many countries, inclusion of only those published in English is a limitation. Also, a potential limitation is that we did not consider reports or data from national cancer registries unless they have been published as peer-reviewed studies and reported trend estimates of yCRC incidence and/or prevalence, in line with our inclusion criteria. The majority of included studies from the USA using potentially overlapping SEER data may also be considered a limitation. However, drawing more nuanced information on yCRC such as sex- and ethnicity-specific trends is only possible with consideration of all included studies; we did not attempt to exclude studies based on potential overlapping data but rather, conducted various sensitivity analyses, which showed no appreciable differences in pooled APCi.

## Conclusion

Overall, by synthesizing findings from peer-reviewed, epidemiologic studies, our systematic review provides empirical evidence that confirms the increased incidence of yCRC, particularly in North America and Australia. Continued efforts for awareness and education to address this increasing risk are warranted along with research to explain this risk so as to identify areas for prevention and intervention.

## Supplementary information

**Additional file 1: Figure S1** Forest plot for studies reporting overall incidence rates for yCRC. **Figure S2** Forest plot for studies reporting incidence rates for yCRC among. **Figure S3** Forest plot for studies reporting incidence rates for yCRC among men

**Additional file 2: Table S1**. Database Search Strategy

**Additional file 3: Table S2**. Quality appraisal checklist

**Additional file 4: Table S3**. Reported incidence rates (per 100,000) for yCRC overall and according to sex among included studies

## Data Availability

All data generated or analysed during this study are included in this published article and its supplementary information files.

## Abbreviations

APC: Annual percent change
CRC: Colorectal cancer
yCRC: Young-onset CRC
IARC: International Agency for Research on Cancer
APCi: Annual percent change in incidence
PRISMA: The Preferred Reporting Items for Systematic Reviews and Meta-Analysis
CI: Confidence intervals
USA: United States of America
SEER: Surveillance, Epidemiology, and End Results Program
AAPCi: Average annual percent change in incidence
EAPCi: Estimated annual percent change in incidence
aCRC: Average-onset colorectal cancer
aHR: Adjusted hazard ratio
