# Supplementary material for: Trends in the epidemiology of young-onset colorectal cancer: a worldwide systematic review
Source: BMC Cancer. 2020 Apr 6;20:288. doi: 10.1186/s12885-020-06766-9 (PMC7137305; doi:10.1186/s12885-020-06766-9)
Supplement: Supplementary file 1 — Additional file 1: Figure S1 Forest plot for studies reporting overall incidence rates for yCRC. Figure S2 Forest plot for studies reporting incidence rates for yCRC among. Figure S3 Forest plot for studies reporting incidence rates for yCRC among men [file 12885_2020_6766_MOESM1_ESM.pptx]

## Slide 1
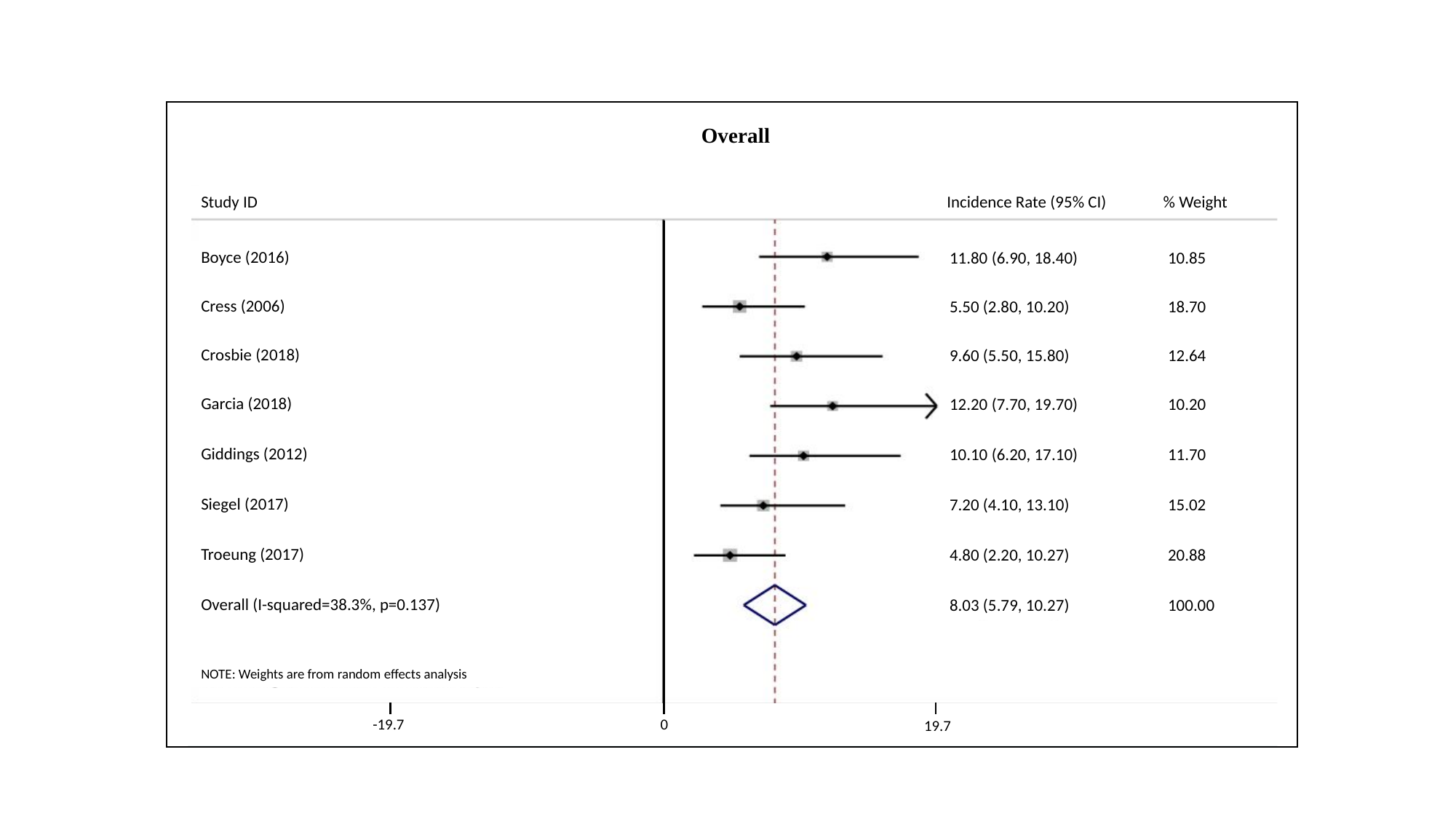

Overall
Incidence Rate (95% CI)
Study ID
% Weight
Study ID						 Incidence Rate (95% CI) % Weight
Boyce (2016)
Cress (2006)
Crosbie (2018)
Garcia (2018)
Giddings (2012)
Siegel (2017)
Troeung (2017)
Overall (I-squared=38.3%, p=0.137)
NOTE: Weights are from random effects analysis
11.80 (6.90, 18.40) 	10.85
5.50 (2.80, 10.20) 	18.70
9.60 (5.50, 15.80) 	12.64
12.20 (7.70, 19.70) 	10.20
10.10 (6.20, 17.10) 	11.70
7.20 (4.10, 13.10) 	15.02
4.80 (2.20, 10.27) 	20.88
8.03 (5.79, 10.27) 	100.00
0
-19.7
19.7

## Slide 2
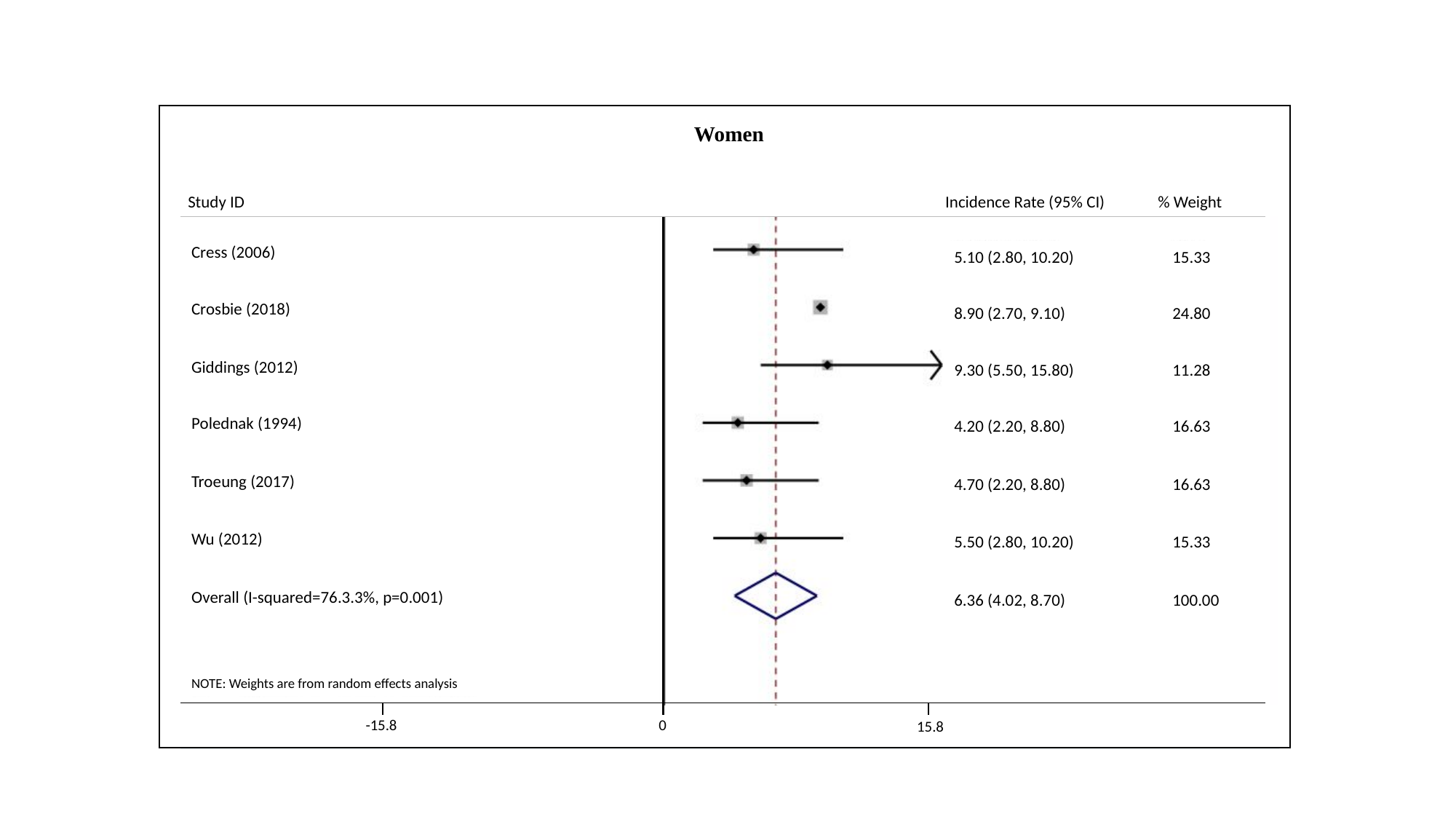

Women
Study ID						 Incidence Rate (95% CI) % Weight
Incidence Rate (95% CI)
% Weight
Cress (2006)
Crosbie (2018)
Giddings (2012)
Polednak (1994)
Troeung (2017)
Wu (2012)
Overall (I-squared=76.3.3%, p=0.001)
NOTE: Weights are from random effects analysis
5.10 (2.80, 10.20) 	15.33
8.90 (2.70, 9.10) 	24.80
9.30 (5.50, 15.80) 	11.28
4.20 (2.20, 8.80) 	16.63
4.70 (2.20, 8.80) 	16.63
5.50 (2.80, 10.20) 	15.33
6.36 (4.02, 8.70) 	100.00
0
-15.8
15.8

## Slide 3
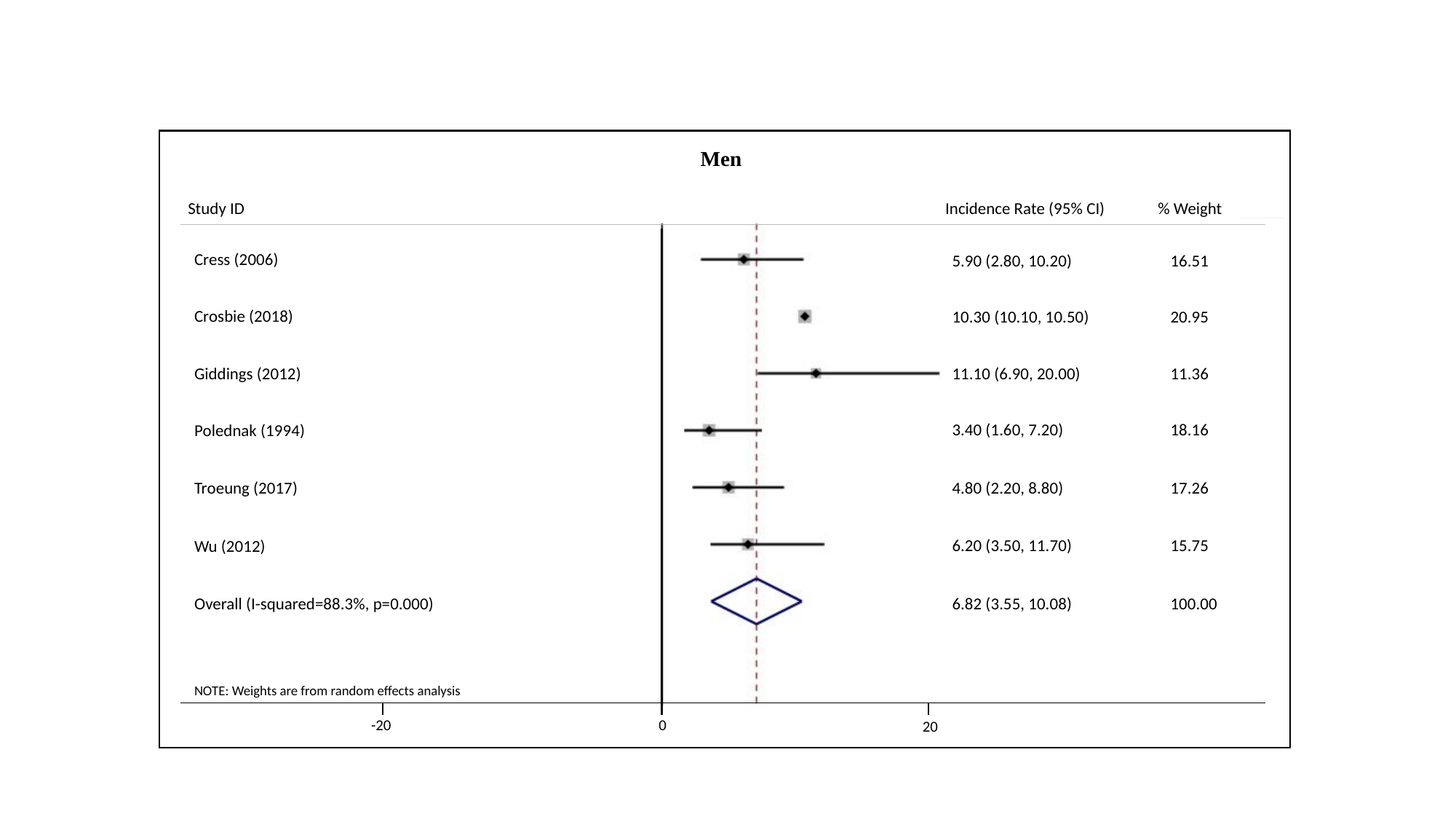

Men
Study ID						 Incidence Rate (95% CI) % Weight
Incidence Rate (95% CI)
% Weight
Incidence Rate (95% CI)
Study ID
% Weight
Cress (2006)
Crosbie (2018)
Giddings (2012)
Polednak (1994)
Troeung (2017)
Wu (2012)
Overall (I-squared=88.3%, p=0.000)
NOTE: Weights are from random effects analysis
5.90 (2.80, 10.20) 	16.51
10.30 (10.10, 10.50) 	20.95
11.10 (6.90, 20.00) 	11.36
3.40 (1.60, 7.20) 	18.16
4.80 (2.20, 8.80) 	17.26
6.20 (3.50, 11.70) 	15.75
6.82 (3.55, 10.08) 	100.00
0
-20
20
