# Supplementary material for: Trends in the epidemiology of young-onset colorectal cancer: a worldwide systematic review
Source: BMC Cancer. 2020 Apr 6;20:288. doi: 10.1186/s12885-020-06766-9 (PMC7137305; doi:10.1186/s12885-020-06766-9)
Supplement: Supplementary file 2 — Additional file 2: Table S1. Database Search Strategy [file 12885_2020_6766_MOESM2_ESM.docx]

**Supplementary Table 1. Database Search Strategy**

| **#** | **Searches** | **Results** |
| --- | --- | --- |
| 1 | exp Colorectal Neoplasms/ | 222458 |
| 2 | colon tumor/ or rectum tumor/ or colorectal tumor/ | 134997 |
| 3 | ("adenomatous polyposis coli" or "adenomatous polyposis colus" or "familial polyposis syndrome*" or "familial adenomatous polyposis coli" or "adenomatous polyposis of the colon" or "familial adenomatous polypos*" or "familial polyposis coli" or "familial adenomatous polyposis of the colon" or "familial multiple polypos*" or "familial polyposis of the colon" or "herary polyposis coli" or "herary polyposis colus" or "familial multiple polypos*" or "familial multiple polyposis syndrome*" or "familial polyposis syndrome*" or "myh associated polypos*" or "polyposis coli" or "polyposis colus" or "familial polyposis colus" or "familial intestinal polypos*" or "adenomatous intestinal polypos*").ti,ab. | 17139 |
| 4 | ("Gardner syndrome*" or "Gardner's syndrome*" or "Gardners syndrome*").ti,ab. | 2264 |
| 5 | ("Lynch cancer family syndrome 2" or "Lynch cancer family syndrome II" or "Lynch syndrome*").ti,ab. | 6712 |
| 6 | ((colorectal or colon or sigmoid or rectal or rectum or anus or anal or perianal or circumanal or rectosigmoid) adj4 (neoplasm* or carcinoma* or cancer* or tumor* or tumour*)).ti,ab. | 449975 |
| 7 | CRC.ti,ab. | 62271 |
| 8 | or/1-7 [CRC] | 544262 |
| 9 | ("young onset" or "early onset" or AYA or "young age" or "younger age" or "early age").ti,ab. | 201499 |
| 10 | (adolescen* or youth* or teen* or "young adult*" or "under 50" or "under the age of 50" or "younger than 50" or "49 and younger" or "15-49 year" or "under 40" or "under the age of 40" or "younger than 40" or "under 30" or "under the age of 30" or "younger than 30" or "under 20" or "under the age of 20" or "younger than 20" or "young patient*" or "younger patient*").ti,ab. | 962686 |
| 11 | 9 or 10 [UNDER 50] | 1138392 |
| 12 | yCRC.ti,ab. | 9 |
| 13 | (8 and 11) or 12 [YOUNG ONSET CRC (focussed)] | 10360 |
| 14 | Incidence/ or cancer incidence/ or incidence*.ti,ab. | 1861525 |
| 15 | Prevalence/ or prevalence*.ti,ab. | 1503427 |
| 16 | Epidemiology/ or Epidemiology.fs. or "cancer epidemiology"/ or epidemiolog*.ti,ab. | 3177339 |
| 17 | or/14-16 [INCID/PREVAL/EPIDEM] | 5195949 |
| 18 | 13 and 17 [YOUNG ONSET CRC (focussed) + INCID/PREVAL/EPIDEM] | 4093 |
| 19 | (English or French or German or Spanish).lg. | 56269666 |
| 20 | 18 and 19 [YOUNG ONSET CRC (focussed) + INCID/PREVAL/EPIDEM with limits] | 3926 |
| 21 | (Animals/ or Animal Experimentation/ or "Models, Animal"/ or (animal* or nonhuman* or non human* or rat or rats or mouse or mice or rabbit or rabbit or pig or pigs or porcine or dog or dogs or hamster or hamsters or fish or chicken or chickens or sheep or cat or cats or raccoon or raccoons or rodent* or horse or horses or racehorse or racehorses or beagle*).ti,ab.) not (Humans/ or (human* or participant* or patient or patients or child* or seniors or adult or adults).ti,ab.) | 8467912 |
| 22 | (editorial or comment or letter or newspaper article).pt. | 3301205 |
| 23 | (conference or conference abstract or congresses).pt. | 3688549 |
| 24 | 20 not (21 or 22 or 23)  [Ovid MEDLINE(R) Epub Ahead of Print, In-Process & Other Non-Indexed Citations, Ovid MEDLINE(R) Daily and Ovid MEDLINE(R) <1946 to Present>](http://ovidsp.tx.ovid.com.ezproxy.library.ubc.ca/sp-3.27.2b/ovidweb.cgi?Titles+Display=G%257CS.sh.593%257C1360&S=EHDMFPOHNDDDAKFONCFKNFGCBPOLAA00)  EBM Reviews - Cochrane Database of Systematic Reviews <2005 to January 10, 2018>  [Embase <1974 to 2018 January 16>](http://ovidsp.tx.ovid.com.ezproxy.library.ubc.ca/sp-3.27.2b/ovidweb.cgi?Titles+Display=G%257CS.sh.593%257C1&S=EHDMFPOHNDDDAKFONCFKNFGCBPOLAA00) | 3077  1718  0  1359 |
| 25 | remove duplicates from 24  [Embase <1974 to 2018 January 16>](http://ovidsp.tx.ovid.com.ezproxy.library.ubc.ca/sp-3.27.2b/ovidweb.cgi?Titles+Display=G%257CS.sh.595%257C1&S=EHDMFPOHNDDDAKFONCFKNFGCBPOLAA00)  [Ovid MEDLINE(R) Epub Ahead of Print, In-Process & Other Non-Indexed Citations, Ovid MEDLINE(R) Daily and Ovid MEDLINE(R) <1946 to Present>](http://ovidsp.tx.ovid.com.ezproxy.library.ubc.ca/sp-3.27.2b/ovidweb.cgi?Titles+Display=G%257CS.sh.595%257C326&S=EHDMFPOHNDDDAKFONCFKNFGCBPOLAA00)  EBM Reviews - Cochrane Database of Systematic Reviews <2005 to January 10, 2018> | 1813  325  1488  0 |
| 26 | Adolescent/ or Young Adult/ | 393686 |
| 27 | Age Factors/ | 851900 |
| 28 | 8 and (26 or 27) [CRC AND (YOUNG OR AGE FACTORS)] | 27142 |
| 29 | 28 and 17 [CRC AND (YOUNG OR AGE FACTORS) + INCID/PREVAL/EPIDEM] | 9934 |
| 30 | 29 and 19 [CRC AND (YOUNG OR AGE FACTORS) + INCID/PREVAL/EPIDEM with limits]  [Ovid MEDLINE(R) Epub Ahead of Print, In-Process & Other Non-Indexed Citations, Ovid MEDLINE(R) Daily and Ovid MEDLINE(R) <1946 to Present>](http://ovidsp.tx.ovid.com.ezproxy.library.ubc.ca/sp-3.27.2b/ovidweb.cgi?Titles+Display=G%257CS.sh.639%257C3165&S=EHDMFPOHNDDDAKFONCFKNFGCBPOLAA00)  [Embase <1974 to 2018 January 16>](http://ovidsp.tx.ovid.com.ezproxy.library.ubc.ca/sp-3.27.2b/ovidweb.cgi?Titles+Display=G%257CS.sh.639%257C1&S=EHDMFPOHNDDDAKFONCFKNFGCBPOLAA00) | 9342  6178  3164 |
| 31 | 30 not (21 or 22 or 23)  [Embase <1974 to 2018 January 16>](http://ovidsp.tx.ovid.com.ezproxy.library.ubc.ca/sp-3.27.2b/ovidweb.cgi?Titles+Display=G%257CS.sh.642%257C1&S=EHDMFPOHNDDDAKFONCFKNFGCBPOLAA00)  [Ovid MEDLINE(R) Epub Ahead of Print, In-Process & Other Non-Indexed Citations, Ovid MEDLINE(R) Daily and Ovid MEDLINE(R) <1946 to Present>](http://ovidsp.tx.ovid.com.ezproxy.library.ubc.ca/sp-3.27.2b/ovidweb.cgi?Titles+Display=G%257CS.sh.642%257C2855&S=EHDMFPOHNDDDAKFONCFKNFGCBPOLAA00) | 8945  2854  6091 |
| 32 | from 31 keep 1-6000 | 6000 |
| 33 | from 31 keep 6001-8945 | 2945 |
| 34 | remove duplicates from 32 | 4664 |
| 35 | remove duplicates from 33 | 2945 |
| 36 | 34 or 35  [Ovid MEDLINE(R) Epub Ahead of Print, In-Process & Other Non-Indexed Citations, Ovid MEDLINE(R) Daily and Ovid MEDLINE(R) <1946 to Present>](http://ovidsp.tx.ovid.com.ezproxy.library.ubc.ca/sp-3.27.2b/ovidweb.cgi?Titles+Display=G%257CS.sh.663%257C2067&S=EHDMFPOHNDDDAKFONCFKNFGCBPOLAA00)  [Embase <1974 to 2018 January 16>](http://ovidsp.tx.ovid.com.ezproxy.library.ubc.ca/sp-3.27.2b/ovidweb.cgi?Titles+Display=G%257CS.sh.663%257C1&S=EHDMFPOHNDDDAKFONCFKNFGCBPOLAA00) | 7609  5543  2066 |
| 37 | 24 or 31 [(yCRC OR GENERAL CRC) + INCID/PREVAL/EPIDEM with limits]  [Embase <1974 to 2018 January 16>](http://ovidsp.tx.ovid.com.ezproxy.library.ubc.ca/sp-3.27.2b/ovidweb.cgi?Titles+Display=G%257CS.sh.672%257C1&S=EHDMFPOHNDDDAKFONCFKNFGCBPOLAA00)  [Ovid MEDLINE(R) Epub Ahead of Print, In-Process & Other Non-Indexed Citations, Ovid MEDLINE(R) Daily and Ovid MEDLINE(R) <1946 to Present>](http://ovidsp.tx.ovid.com.ezproxy.library.ubc.ca/sp-3.27.2b/ovidweb.cgi?Titles+Display=G%257CS.sh.672%257C3813&S=EHDMFPOHNDDDAKFONCFKNFGCBPOLAA00) | 10796  3812  6984 |
| 38 | 37 not 24 [GENERAL CRC NOT FOCUSSED yCRC]  [Embase <1974 to 2018 January 16>](http://ovidsp.tx.ovid.com.ezproxy.library.ubc.ca/sp-3.27.2b/ovidweb.cgi?Titles+Display=G%257CS.sh.681%257C1&S=EHDMFPOHNDDDAKFONCFKNFGCBPOLAA00)  [Ovid MEDLINE(R) Epub Ahead of Print, In-Process & Other Non-Indexed Citations, Ovid MEDLINE(R) Daily and Ovid MEDLINE(R) <1946 to Present>](http://ovidsp.tx.ovid.com.ezproxy.library.ubc.ca/sp-3.27.2b/ovidweb.cgi?Titles+Display=G%257CS.sh.681%257C2454&S=EHDMFPOHNDDDAKFONCFKNFGCBPOLAA00) | 7719  2453  5266 |
| 39 | from 38 keep 1-6000 | 6000 |
| 40 | from 38 keep 6001-7719 | 1719 |
| 41 | remove duplicates from 39 | 4587 |
| 42 | remove duplicates from 40 | 1719 |
| 43 | 41 or 42  [Ovid MEDLINE(R) Epub Ahead of Print, In-Process & Other Non-Indexed Citations, Ovid MEDLINE(R) Daily and Ovid MEDLINE(R) <1946 to Present>](http://ovidsp.tx.ovid.com.ezproxy.library.ubc.ca/sp-3.27.2b/ovidweb.cgi?Titles+Display=G%257CS.sh.696%257C1629&S=EHDMFPOHNDDDAKFONCFKNFGCBPOLAA00)  [Embase <1974 to 2018 January 16>](http://ovidsp.tx.ovid.com.ezproxy.library.ubc.ca/sp-3.27.2b/ovidweb.cgi?Titles+Display=G%257CS.sh.696%257C1&S=EHDMFPOHNDDDAKFONCFKNFGCBPOLAA00) | 6306  4678  1628 |

**EBSCOhost CINAHL Complete**

1937-2018

| **#** | **Searches** | **Results** |
| --- | --- | --- |
| 1 | (MH "Colorectal Neoplasms+") | (29,768) |
| 2 | TI ("adenomatous polyposis coli" or "adenomatous polyposis colus" or "familial polyposis syndrome*" or "familial adenomatous polyposis coli" or "adenomatous polyposis of the colon" or "familial adenomatous polypos*" or "familial polyposis coli" or "familial adenomatous polyposis of the colon" or "familial multiple polypos*" or "familial polyposis of the colon" or "herary polyposis coli" or "herary polyposis colus" or "familial multiple polypos*" or "familial multiple polyposis syndrome*" ... | (512) |
| 3 | TI ("Gardner syndrome*" or "Gardner's syndrome*" or "Gardners syndrome*") OR AB ("Gardner syndrome*" or "Gardner's syndrome*" or "Gardners syndrome*") | (55) |
| 4 | TI ("Lynch cancer family syndrome 2" or "Lynch cancer family syndrome II" or "Lynch syndrome*") OR AB ("Lynch cancer family syndrome 2" or "Lynch cancer family syndrome II" or "Lynch syndrome*") | (461) |
| 5 | TI ((colorectal or colon or sigmoid or rectal or rectum or anus or anal or perianal or circumanal or rectosigmoid) N4 (neoplasm* or carcinoma* or cancer* or tumor* or tumour*)) OR AB ((colorectal or colon or sigmoid or rectal or rectum or anus or anal or perianal or circumanal or rectosigmoid) N4 (neoplasm* or carcinoma* or cancer* or tumor* or tumour*)) | (28,447) |
| 6 | TI (CRC) OR AB (CRC) | (4,142) |
| 7 | S1 OR S2 OR S3 OR S4 OR S5 OR S6 | (39,050) |
| 8 | TI ("young onset" or "early onset" or AYA or "young age" or "younger age" or "early age") OR AB ("young onset" or "early onset" or AYA or "young age" or "younger age" or "early age") | (16,876) |
| 9 | TI (adolescen* or youth* or teen* or "young adult*" or "under 50" or "under the age of 50" or "younger than 50" or "49 and younger" or "15-49 year" or "under 40" or "under the age of 40" or "younger than 40" or "under 30" or "under the age of 30" or "younger than 30" or "under 20" or "under the age of 20" or "younger than 20" or "young patient*" or "younger patient*") OR AB (adolescen* or youth* or teen* or "young adult*" or "under 50" or "under the age of 50" or "younger than 50" or "49 and you ... | (156,648) |
| 10 | S8 OR S9 | (170,619) |
| 11 | S7 AND S10 | (682) |
| 12 | TI (yCRC) OR AB (yCRC) | (1) |
| 13 | S11 OR S12 | (682) |
| 14 | (MH "Incidence") | (50,729) |
| 15 | (MH "Prevalence") | (67,800) |
| 16 | (MH "Epidemiology") | (5,149) |
| 17 | TI (incidence* OR prevalence* OR epidemiolog*) OR ab (incidence* OR prevalence* OR epidemiolog*) | (70,017) |
| 18 | S14 OR S15 OR S16 OR S17 | (156,147) |
| 19 | S13 AND S18 | (103) |
| 20 | ((MH "Vertebrates+") NOT MH Human) | (150,281) |
| 21 | S19 NOT S20 | (102) |
| 22 | PT ("letter" OR "commentary" OR "editorial" OR "letter" OR "letter to the or" OR "newspaper" OR "pamphlet" OR "pamphlet chapter") | (608,504) |
| 23 | PT ("conference paper" OR "conference proceeding") | (2,426 |
| 24 | S21 NOT S22 | (99) |
| 25 | S24 NOT S23 | (99) |
| 26 | LA ("english" OR "french" OR "german" OR "spanish) | (5,775,987) |
| 27 | S25 AND S26 [YOUNG ONSET CRC (focussed) + INCID/PREVAL/EPIDEM with limits] | (99) |
| 28 | MH ("Adolescence" OR "Young Adult") | (490,492) |
| 29 | MH "Age Factors" | (98,322) |
| 30 | S7 AND (S28 OR S29) | (2,618) |
| 31 | S30 AND S18 | (354) |
| 32 | S31 NOT S20 | (353) |
| 33 | S32 NOT S22 | (345) |
| 34 | S33 NOT S23 | (345) |
| 35 | S34 AND S26 | (343) |
| 36 | S35 NOT S27 [CRC AND (YOUNG OR AGE FACTORS) + INCID/PREVAL/EPIDEM with limits, excluding yCRC (focused)] | (290) |
