# Supplementary material for: Trends in the epidemiology of young-onset colorectal cancer: a worldwide systematic review
Source: BMC Cancer. 2020 Apr 6;20:288. doi: 10.1186/s12885-020-06766-9 (PMC7137305; doi:10.1186/s12885-020-06766-9)
Supplement: Supplementary file 3 — Additional file 3: Table S2. Quality appraisal checklist [file 12885_2020_6766_MOESM3_ESM.docx]

**Supplementary Table 2. Quality appraisal checklist**

| **Item** | **Yes**  **(=1)** | **No (=0)** | **Unclear (=0)** | **NA (=0)** |
| --- | --- | --- | --- | --- |
| **Introduction** (1 item) |  |  |  |  |
| 1. Were the aims/objectives of the study clear?^*^ |  |  |  |  |
| **Methods** (14 items) |  |  |  |  |
| 2. Was the study design appropriate for the stated aim(s)?^*^ |  |  |  |  |
| 3. Is the sample size adequate?^§^ |  |  |  |  |
| 4. Were the study subjects and setting described in detail?^§^ |  |  |  |  |
| 5. Was the sample frame taken from an appropriate population base so that it closely represented the target/reference population under investigation?^*^ |  |  |  |  |
| 6. Was the selection process likely to select subjects/participants that were representative of the target/reference population under investigation?^*^ |  |  |  |  |
| 7. Were objective, standard criteria used for the measurement of the condition?^§^ |  |  |  |  |
| 8. Was the condition measured reliably?^§^ |  |  |  |  |
| 9. Were the risk factor and outcome variables measured appropriate to the aims of the study?^*^ |  |  |  |  |
| 10. Were the risk factor and outcome variables measured correctly using instruments/measurements that had been trialed, piloted or published previously?^*^ |  |  |  |  |
| 11. Was the data analysis conducted with sufficient coverage of the identified sample?^§^ |  |  |  |  |
| 12. Was there appropriate statistical analysis? ^§^ |  |  |  |  |
| 13. Is it clear what was used to determined statistical significance and/or precision estimates? (eg, p values, CIs)?^*^ |  |  |  |  |
| 14. Were the methods (including statistical methods) sufficiently described to enable them to be repeated?^*^ |  |  |  |  |
| 15. Are all important confounding factors/subgroups/differences identified and accounted for? ^§^ |  |  |  |  |
| **Results** (3 items) |  |  |  |  |
| 16. Were the basic data adequately described? ^*^ |  |  |  |  |
| 17. Were the results internally consistent? ^*^ |  |  |  |  |
| 18. Are the estimates of prevalence or incidence given with confidence intervals and in detail by subgroup, if appropriate? ^*^ |  |  |  |  |
| **Discussion** (2 items) |  |  |  |  |
| 19. Were the authors’ discussions and conclusions justified by the results? ^*^ |  |  |  |  |
| 20. Were the limitations of the study discussed? ^*^ |  |  |  |  |
| **Total score** |  | | | |

^§^Item from the Joanna Briggs Institute Prevalence Critical Appraisal Tool;

*Item from the AXIS tool
