# Supplementary material for: Trends in the epidemiology of young-onset colorectal cancer: a worldwide systematic review
Source: BMC Cancer. 2020 Apr 6;20:288. doi: 10.1186/s12885-020-06766-9 (PMC7137305; doi:10.1186/s12885-020-06766-9)
Supplement: Supplementary file 4 — Additional file 4: Table S3. Reported incidence rates (per 100,000) for yCRC overall and according to sex among included studies [file 12885_2020_6766_MOESM4_ESM.docx]

**Supplementary Table 3. Reported incidence rates (per 100,000) for yCRC overall and according to sex among included studies**

| **Study** | **Date Range** | **Overall** | **Women** | **Men** |
| --- | --- | --- | --- | --- |
| **Studies reporting overall** | | | | |
| Meyer, 2010 | 1973-2005 | CC: 1.11  RC: 0.42 | - | - |
| Boyce, 2016 | 2001 to 2008 | CRC: 11.8 | - | - |
| Siegel, 2017 | 2004-2013 | CRC: 7.2 | - | - |
| Ansa, 2018 | 2000-2014 | CRC <40y: 2.3 (2.3, 2.4)  CRC 40-49y: 22.5 (22.1, 22.8) | - | - |
| Garcia, 2018 | 2001-2014 | CRC: 12.2 | - | - |
| Ullah, 2018 | 1994-2012 | CRC 20-29y: 0.0291  CRC 30-39y: 0.0634  CRC 40-49y: 0.2066 | - | - |
| **Studies reporting according to sex** | | | | |
| Zaridze, 1990 | 1971 to 1987 | - | CC <29 y: 0.2  CC 30-39y: 2.8  CC 40-49 y: 9.5  RC <29 y: 0.1  RC 30-39y: 0.8  RC 40-49 y: 8.0 | CC <29 y: 0.2  CC 30-39y: 1.9  CC 40-49 y: 8.2  RC <29 y: 0.1  RC 30-39y: 1.3  RC 40-49 y: 7.5 |
| Chow, 1991 | 1976-1987 | - | CC White: 4.2  CC Black: 6.3 | CC White: 4.7  CC Black: 7.2 |
| Polednak, 1994 | 1965-1991 | - | CRC: 4.2 | CRC: 3.4 |
| Giddings, 2012 | 1998-2007 | CRC: 10.1* | CRC: 9.3*  CRC Chinese: 4.3  CRC Japanese: 4.4  CRC Filipino: 4.5  CRC Korean: 5.3  CRC South Asian: 3.8  CRC Vietnamese: 6.0 | CRC: 11.1*  CRC Chinese: 4.3  CRC Japanese: 6.0  CRC Filipino: 5.1  CRC Korean: 9.7  CRC South Asian: 4.2  CRC Vietnamese: 6.8 |
| Wu, 2012 | 1973 to 2005 | - | CRC: 5.5 | CRC: 6.2 |
| Gandhi, 2017 | 1975 to 2012 | - | proximal CC: 1.9  distal CC: 2.1  RC: 2.3 | proximal CC: 1.3  distal CC: 1.8  RC: 2.9 |
| Ellis, 2018 | 2010-2014 | - | CRC Chinese: 3.3 (2.5, 4.3)  CRC Japanese: 4.7 (2.9, 7.3)  CRC Filipino: 2.8 (2.1, 3.8)  CRC Korean: 2.7 (1.6, 4.4)  CRC South Asian: 2.6 (1.5, 4.1)  CRC Vietnamese: 3.6 (2.3, 5.2)  CRC SEast Asian: 1.7 (0.6, 3.9)  CRC White: 4.0 (3.6, 4.3)  CRC Black: 3.9 (3.1, 4.8)  CRC Hispanic: 3.0 (2.7, 3.3) | CRC Chinese: 3.2 (2.4, 4.4)  CRC Japanese: 5.0 (2.8, 8.2)  CRC Filipino: 3.6 (2.7, 4.8) CRC Korean: 4.7 (3.0, 7.0)  CRC South Asian: 2.4 (1.4, 3.7)  CRC Vietnamese: 4.0 (2.7, 5.8)  CRC SEast Asian: 3.7 (1.7, 6.9)  CRC White: 3.8 (3.5, 4.1)  CRC Black: 4.0 (3.2, 4.8)  CRC Hispanic: 2.8 (2.5, 3.0) |
| **Studies reporting overall and according to sex** | | | | |
| Cress, 2006 | 1992-2001 | CRC: 5.5* | CRC: 5.1 | CRC: 5.9 |
| Wang, 2017 | 1995-2010 | CRC 20-39y: 3.7  CRC 40-49y: 20.0 | CRC 20-39: 3.7  CRC 40-49y: 17.1 | CRC 20-39: 3.8  CRC 40-49y: 22.9 |
| Troeung, 2017 | 1982 to 2007 | CRC: 4.8 | CRC: 4.7 | CRC: 4.8 |
| Crosbie, 2018 | 1992-2014 | CRC: 9.6 (9.5, 9.8) | CRC: 8.9 (8.7, 9.1) | CRC: 10.3 (10.1, 10.5) |

Where incidence rates were reported for more than one time period, we tabulated those for the most recent;

p-values corresponding to incidence rates tabulated as reported;

*****- obtained from authors after contacting them;

**Abbreviations**: CRC – colorectal cancer; RC – rectal cancer; CC – colon cancer;
